# Characterization of Betulinic Acid-Multiwalled Carbon Nanotubes Modified with Hydrophilic Biopolymer for Improved Biocompatibility on NIH/3T3 Cell Line

**DOI:** 10.3390/polym13091362

**Published:** 2021-04-21

**Authors:** Julia Meihua Tan, Saifullah Bullo, Sharida Fakurazi, Mohd Zobir Hussein

**Affiliations:** 1 Materials Synthesis and Characterization Laboratory, Institute of Advanced Technology (ITMA), Universiti Putra Malaysia, Serdang 43400, Selangor, Malaysia; juliatan@xmu.edu.my (J.M.T.); saifullah.bullo@bnbwu.edu.pk (S.B.); 2 New Energy Science and Chemical Engineering, Xiamen University Malaysia, Jalan Sunsuria, Bandar Sunsuria, Sepang 43900, Selangor, Malaysia; 3 Department of Management Science & Technology, The Begum Nusrat Bhutto Women University, Sukkur 65170, Sindh, Pakistan; 4 Department of Human Anatomy, Faculty of Medicine and Health Sciences, Universiti Putra Malaysia, Serdang 43400, Selangor, Malaysia; sharida@upm.edu.my

**Keywords:** hydrophilic polymer, oxidized nanotubes, cytotoxicity, NIH/3T3 cells, nanomedicine

## Abstract

The biocompatibility of carbon nanotubes (CNT) is fairly a challenging task for their applications in nanomedicine. Therefore, the objective of this research was to formulate four types of highly biocompatible betulinic acid-loaded biopolymer nanocomposites, namely chitosan-multiwalled carbon nanotubes (MWBA-CS), polyethylene glycol-multiwalled carbon nanotubes (MWBA-PG), Tween 20-multiwalled carbon nanotubes (MWBA-T2) and Tween 80-multiwalled carbon nanotubes (MWBA-T8). The physico-chemical properties of the modified nanocomposites were determined by Fourier transform infrared spectroscopy (FTIR), thermal analysis (TGA) and Raman spectroscopy, while the surface morphology of the resulting nanocomposites was studied using field emission scanning electron microscopy (FESEM). All data revealed that the external surface of MWBA nanocomposites was successfully coated with the respective polymer molecules through hydrophobic and electrostatic interactions with improved thermal profiles. The cell viability assay, which was performed on cultured normal embryonic mouse fibroblast cells, confirmed their excellent biocompatibility in phosphate-buffered saline aqueous media. Overall, our findings herein suggest that the synthesized biopolymer-coated MWBA nanocomposites are promising nanomaterials for drug delivery applications as they enhance the solubility and dispersibility of CNT with significantly reduced cytotoxic effect, especially in normal cells.

## 1. Introduction

The concept of nanoscience was first introduced by a Nobel Laureate Caltech physicist, Richard Feynman, in his after-dinner speech entitled “There’s plenty of room at the bottom”, which was later published in 1960 [1]. In this speech, Feynman addressed the significance of maneuvering materials on a sub-microscopic level as individual atoms could behave differently and that they do not scale down in proportion. Later, this concept was coined as nanotechnology by a Japanese scientist, Norio Taniguchi, in 1974 at an international scientific conference on precision manufacturing technology that described materials with tolerances of a micron or less [2]. The prefix “nanos” is a Greek word meaning dwarf, and one nanometer is a length scale that is equivalent to one billionth of a meter. 

Since ancient times, natural products derived from botanical sources have typically been used as traditional remedies to treat sore throats, toothache, colds and influenza due to their abundant availability and exceptional chemical and biological features, with macromolecular specificity [3]. Betulinic acid (BA) is a natural phyto-compound mainly isolated from the stem bark of white birch and other tree species widely distributed throughout the tropics. According to research, the pentacyclic triterpene with a lupane structure and its derivative compounds demonstrate potent cytotoxicity for skin cancer cells [4,5] and human immunodeficiency virus [6], while displaying several other pharmacological properties such as anti-bacterial, anti-inflammatory, anti-nociceptive, anti-malarial and anti-helminthic activities [7]. Despite all these advantages, concerns related to issues such as the toxicity of solvent residue, short half-life and low bioavailability due to poor aqueous solubility in the human body present a greater challenge of using BA as a medicine for disease prevention and treatment. To overcome this barrier, the application of nanotechnology in medical therapeutics (this has been termed nanomedicine) can be regarded as a novel and state-of-the-art solution for enhancing the efficacy of a poorly water-soluble drug such as BA, with the utilization of smart nanoscale material (fullerenes, metal particles, carbon nanotubes, organic and inorganic nanoparticles, etc.) acting as the promising drug delivery agent. 

Among these nanoscale agents, nanomaterials such as carbon nanotubes (CNT), which possess unique physico-chemical properties, have been extensively researched by scientists in recent years for cancer diagnosis [8], cancer immunotherapy [9], targeted drug delivery [10] and biosensors [11] for their potential applications in nanomedicine. CNT, discovered in 1991 by Japanese scientist Iijima [12], are allotropes of carbon made of graphene sheets rolled into nanosized cylindrical tubes with several micrometers in length and nanometer scale in diameter. Based on the number of rolled graphene layers, CNT can be further classified into two main groups: single walled CNT (SWNT) and multiwalled CNT (MWNT). Although these nanomaterials are lightweight, they possess highly conductive electrical and thermal properties while demonstrating ultimate mechanical tensile strength in the range of 100–200 GPa, which is about 100 times stronger than that of steel of the same diameter [13]. 

In recent years, CNT have been successfully applied as a promising drug delivery vehicle in nanomedicine due to their high specific surface area (internally and externally) and rich electronic polyaromatic structure, which is capable of conjugating with numerous types of therapeutic agents, for example, drugs [10,14], proteins [15], antibodies, [16] genes [17] and vaccines [18] for targeting drug delivery. In addition, oxidized CNT have been reported to display anti-microbial activity against various pathogenic bacteria [19], and they can also be utilized as a potential anti-oxidant in free radical scavenging owing to the presence of carboxylic acid (–COOH) functional groups [20]. One of the most interesting features of these nanomaterials is that they are capable of internalizing into mammalian cells by endocytosis and traveling directly into the cytoplasm and potentially the nucleus [21] for drug delivery, owing to the π-π stacking and hydrophobic interaction as well as surface electrostatic adsorption. On top of that, they can keep the bioactive drug molecules intact during translocation and cellular penetration without being rapidly metabolized and excreted by the human body [22], and thus further prevent the degradation of the drug. Subsequently, this allows the drug, particularly cytotoxic chemotherapy drugs, to be used at lower dosages and further minimizes the toxic side effects, resulting in greater patient compliance. 

With the rapid development of nanomedicine in the 21st century, there have been growing concerns about CNT’s possible cytotoxicity that can induce apoptotic cell death and cause mitochondrial impairment through the overproduction of reactive oxygen species [23]. Despite the cellular toxicity, the immunological alterations caused by CNT-based nanomaterials, for example, pulmonary macrophage activation and inflammation induced by the activation of the complement system, can be another potential detrimental concern [24]. The complication of the CNT toxicity issue is contributed by several factors such as shape, length, diameter, purity, surface area and surface functional groups of the nanotubes [25]. When considering their biomedical applications in vitro, the main hindrances are their tendency to form agglomerates in the physiological environment resulting from aggregation due to strong van der Waals or electrostatic forces between the nanotubes. This generates an urgent need to adopt alternative methods such as ultra-sonication, vigorous agitation, surface chemical treatments, the addition of surfactants or incorporation with water-soluble polymers to improve the overall solubility effect of CNT in aqueous condition. 

To address the unmet pharmaceutical needs, we have employed the latter method by using water-soluble polymers (e.g., chitosan and polyethylene glycol) and non-ionic surfactants (e.g., Tween 20 and Tween 80) as the coating agents for our MWNT loaded with BA formulation (herein designated as MWBA). These biopolymers are well known for their intrinsic hydrophilicity, good stability, non-toxicity to the environment and excellent biocompatibility, as well as being commercially inexpensive. They are typically used in numerous biomedical, pharmaceutical and food applications, for example, cartilage tissue engineering [26], wound healing [27], drug delivery [28], food matrix [29] and many more. In addition, the combination of CNT (inorganic component) and biomaterial (organic component) in the administration of cancer immunotherapy may complement each other to promote synergistic effects through enhanced immune reactions of the body to attack the cancer cells [9]. To the best of our knowledge, research related to the biocompatibility of these biopolymers is not sufficiently documented in the literature. Therefore, the biological and safety profiles of these molecules need to be extensively characterized and understood prior to their utilization in the treatment of cancer therapy.

In our previous study, we successfully synthesized an MWBA formulation where it displayed a much more potent anti-cancer activity against the human lung A549 carcinoma cell line and the human liver HepG2 carcinoma cell line than the BA drug alone [30]. However, the cell viability test of the studied material for normal and healthy mouse embryo fibroblast cells showed a much higher toxicity profile at dosages above 50 μg/mL. Due to this reason, the primary aim of this work was to assess and compare the cytotoxic effects of the biopolymer-coated MWBA formulations on non-tumor fibroblast cell line NIH/3T3 as the preliminary in vitro toxicity screening using MTT ([3-(4,5-dimethylthiazol-2-yl)-2,5-diphenyltetrazolium bromide]) colorimetric assay. The nanocomposites were prepared non-covalently through the integration of short, oxidized MWNT loaded with BA into a polymer matrix core shell. The obtained nanocomposites were then subjected to vigorous physico-chemical characterizations and a cell viability biological test. 

## 2. Materials and Methods 

### 2.1. Materials

Short, oxidized MWNT (MWNT-COOH) produced by chemical vapor deposition was bought from Chengdu Organic Chemicals Co. Ltd. (Chengdu, China) with outside diameter 20–30 nm, length 0.5–2.0 μm, purity >95 wt%, COOH functional content 1–2 wt% and ash content <1.5 wt%. Betulinic acid (BA, 90% purity) with molecular formula C_30_H_48_O_3_, Tween 20 (T2), Tween 80 (T8) and partially N-deacetylated chitosan (CS, deacetylation degree = 75–85%, medium molecular weight = 190–310 kDa) were procured from Sigma Aldrich (Saint Louis, MI, USA). Polyethylene glycol (PG) with an average molecular weight of 300 kDa was obtained from Acros Organics (Geel, Belgium). Methanol and acetic acid (both 99.8% purity) were sourced from HmbG Chemicals (Hamburg, Germany). All other chemicals and reagents used were of analytical grade.

### 2.2. BA Loading onto MWNT-COOH 

The MWBA was prepared following the non-covalent method described previously [30]. BA solution was obtained by dissolving the drug in methanol (0.125 mg/mL) through stirring and heating to around 37–40 °C until all BA had fully dissolved. Then, 400 mg of oxidized MWNT was dispersed in BA solution by ultra-sonication treatment for approximately 1 h to mechanically isolate the bundled nanotubes and enhance their solubility. Subsequently, the suspension was reacted under constant vigorous stirring at room temperature and maintained at pH 4 for optimum drug loading. After stirring for 22 h, the mixture was then filtered, centrifuged, washed simultaneously with methanol and deionized water three times and finally dried in an oven at 60 °C overnight. Later, it was allowed to cool to an ambient temperature to yield MWBA. The sample was homogeneously powdered and kept in an airtight bottle for further use. 

### 2.3. Surface Coating of Hydrophilic Biopolymer onto MWBA

Preparation of biocompatible polymeric MWBA nanocomposites was carried out according to the literature with slight modifications [31]. Briefly, 100 mg of synthesized BA-loaded nanotubes were added to 1% T2 dissolved into 100 mL of deionized water. After that, the mixture was subjected to constant-stirring using a magnetic stirrer at room temperature for 24 h. The black residue was then collected, centrifuged and washed with deionized water three times to remove excessive T2 which was not reacted in the coating process. The resulting product was obtained after drying completely in an oven at 60 °C overnight. The rest of the biopolymeric MWBA nanocomposites were formulated using a similar method except for 0.5% CS solution which was prepared by dissolving the CS flakes into 100 mL of aqueous acetic acid under constant vigorous stirring at room temperature. The samples were denoted as MWBA-T2, MWBA-T8, MWBA-PG and MWBA-CS.

### 2.4. UV-vis Spectrophotometry

The supernatant containing unreacted BA was collected and subjected to spectrophotometric analysis. The determination of BA loading efficiency onto MWNT-COOH was quantified using a Perkin Elmer Lambda 35 UV-vis spectrophotometer (USA). The samples were characterized at a wavelength of 209 nm (the absorption peak of BA) and the measurement was performed with a quartz cuvette of path length 1 cm. The drug loading efficiency was estimated to be approximately 15% using the following equation:

(1) $$\mathrm{Drug}\;\mathrm{loading}\;\mathrm{efficiency}\;\%\;=\;\frac{\left(\mathrm{Initial}\;\mathrm{amount}\;\mathrm{of}\;\mathrm{BA}\;-\;\mathrm{Amount}\;\mathrm{of}\;\mathrm{unreacted}\;\mathrm{BA}\right)}{\left(\mathrm{Initial}\;\mathrm{amount}\;\mathrm{of}\;\mathrm{BA}\right)}\;\times 100$$

### 2.5. Fourier Transform Infrared Spectroscopy

The chemical structures of MWBA and polymeric MWBA nanocomposites were investigated using a Thermo Nicolet Nexus Smart Orbit FTIR spectrophotometer (USA). The FTIR spectra of the samples were taken in the wavenumber region between 4000 and 400 cm^−1^ using the KBr pellet technique except for T2 and T8 by a direct deposition technique. To obtain a single spectrum, a total of 32 scans at a resolution of 4 cm^−1^ were accumulated using a rapid-scan software in OMNIC 8.0. Prior to measurements, all samples were dried in an oven at 60 °C for 24 h.

### 2.6. Thermogravimetric Analysis

The thermal profiles of the samples were recorded using a TA Q500 thermogravimetric analyzer (USA). The samples were scanned over a temperature range from room temperature to 1000 °C at a heating rate of 10 °C/min under a nitrogen dynamic flow rate of 40 mL/min. To estimate the wt% of the biopolymer coated onto MWBA, thermogravimetric analysis (TGA) was also performed on blank MWNT-COOH coated with the respective hydrophilic biopolymers. A set of four samples designated as MWNT-CS, MWNT-PG, MWNT-T2 and MWNT-T8 were synthesized identically using a similar method as described in Section 2.3 above. The estimated biopolymer content in the samples was found to be around 8.01, 9.13, 18.96 and 59.18 wt% for MWNT-CS, MWNT-PG, MWNT-T2 and MWNT-T8, respectively, as compared to the blank MWNT-COOH. All samples were dried at 60 °C for 24 h to remove the moisture adsorbed on the surface of the samples before carrying out the analysis.

### 2.7. Raman Spectroscopy

Raman analysis was performed on a WITec confocal Raman microscopy system Alpha 300R (Germany) equipped with a UHTS-300 spectrometer using 532 nm excitation wavelength. The samples were ultra-sonicated for 10 min at room temperature and several drops from the suspension were placed on a glass slide prior to the measurements. Raman detailed scans were carried out within the wavenumber range 100–2000 cm^−1^. 

### 2.8. Field Emission Scanning Electron Microscopy

The surface morphology of the samples was observed by the JEOL JSM-7600F and Fei Tecnai G20 field emission scanning electron microscopes operated at an accelerating voltage of 5 and 10 kV. The sample was previously dried and ground before being placed in an aluminum sample holder secured on adhesive tape. The sample was then coated with a thin layer of gold using a sputter coater. 

### 2.9. Determination of Cytotoxicity Properties of Biopolymeric MWBA Nanocomposites

The in vitro cell viability was conducted on NIH/3T3 cells, a normal and healthy mouse embryonic fibroblast cell line obtained from ATCC using the standard MTT assay. The cells were cultured in a fresh medium containing RPMI supplemented with 10% fetal bovine serum and 1% penicillin-streptomycin in a 5% CO_2_ atmosphere and 95% relative humidity at 37 °C for a period of 24 h. When the confluency of cells reached around 80%, the cell line was then transferred and sub-cultured in a new culture flask for seeding and treatment purposes using 0.25% trypsin-EDTA solution. Stock solutions containing biopolymeric MWBA nanocomposites were freshly prepared in PBS and diluted serially to obtain a series of concentrations ranging from 1.56 μg/mL to 100 μg/mL. The cells cultured without nanocomposites were used as a control group (cells in media only). 

For cytotoxicity assay, NIH/3T3 cells were seeded (10,000 cells/well) in a 96-well microplate and maintained for 1 day at 37 °C (5% CO2 and 95% air) to allow for cell attachment. The cells were exposed to different concentrations of biopolymeric MWBA nanocomposites for 72 h and further incubated with MTT solution (20 μL, 5 mg/mL in PBS) for another 3 h. Thereafter, excessive MTT reagent was discarded and the purple formazan crystals formed in the assay were solubilized with DMSO (200 μL). The absorption was recorded at 570 nm against 630 nm as a reference wavelength using an EL 800X microplate reader (Winooski, VT, USA). The following formula was used to determine the absorbance value which is directly proportional to the cell viability. All MTT assays were repeated in triplicate.

(2) $$\mathrm{Cell}\;\mathrm{Viability}\;\%\;=\;\frac{\left(A_{\mathrm{test}}-{\;A}_{\mathrm{blank}\;}\right)}{\left(A_{\mathrm{control}}\;-{\;A}_{\mathrm{blank}}\right)}\;\times 100$$

where A_test_ is the absorbance of the cells treated with biopolymeric MWBA nanocomposites, A_blank_ is the absorbance from empty wells and A_control_ refers to the absorbance of the cells without biopolymeric MWBA nanocomposites.

### 2.10. Statistical Analysis

Each measurement was performed using at least three fresh, independently prepared samples (*n* = 3) and the results were expressed as the mean ± standard deviation. The data were subjected to one-way analysis of variance (ANOVA) using the SPSS version 25.0 statistical software package (Armonk, NY, USA). Tukey HSD post-hoc test was also applied to determine the significant difference (* *p* <0.05) between the biopolymeric MWBA nanocomposites using a 95% confidence interval. 

## 3. Results and Discussion

### 3.1. FTIR Spectra of MWBA Coated with Biopolymers

**Figure 1:**
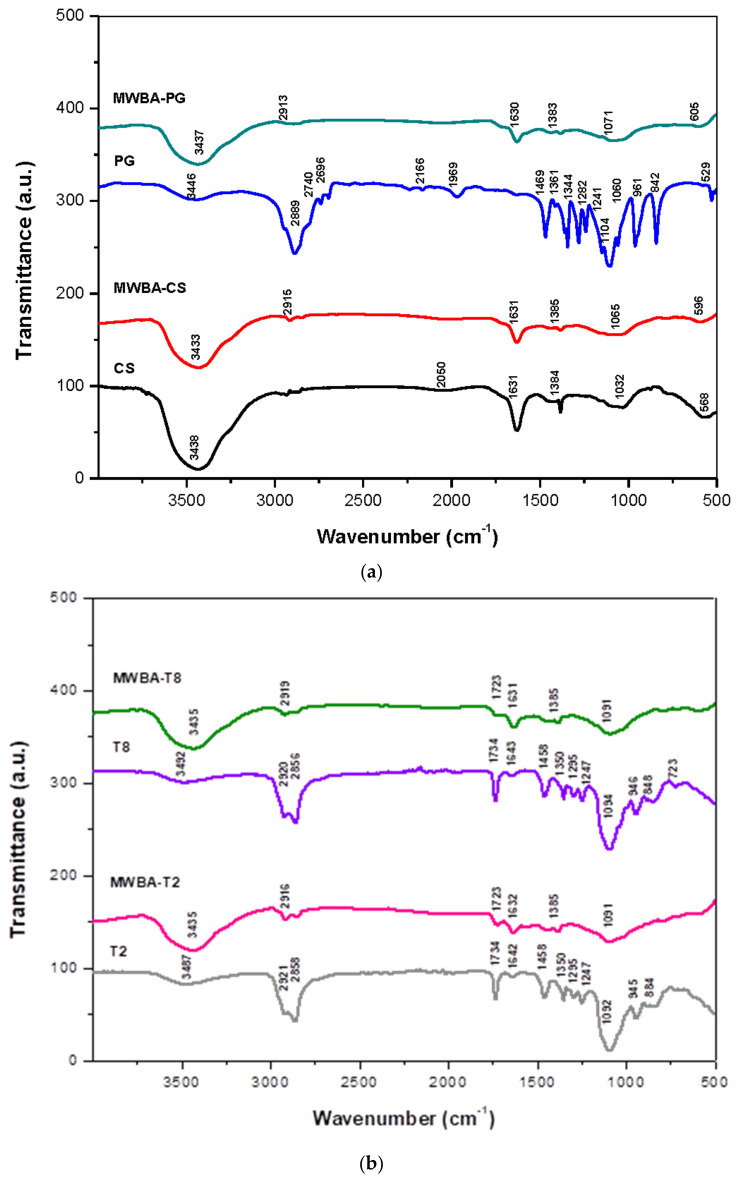
FTIR spectra of samples: (**a**) chitosan (CS), MWBA-CS, polyethylene glycol (PG), MWBA-PG and (**b**) Tween 20 (T2), MWBA-T2, Tween 80 (T8) and MWBA-T8.

FTIR spectroscopy is a quantitative analytical tool widely used in many research applications as one of the possible ways to elucidate the chemical structure and molecular interaction among the compounds present in a sample [32]. We have reported the FTIR spectra of the blank MWNT–COOH and the BA-loaded nanotubes extensively in our previous paper [30]. Hence, in the current study, we focus on the possible interaction between the functional groups of the biopolymers and BA-loaded nanotubes. Figure 1 illustrates the FTIR spectra of the (a) CS, MWBA-CS, PG, MWBA-PG and (b) T2, MWBA-T2, T8 and MWBA-T8. Generally, the characteristic bands that occurred at 2919–2913 cm⁻^1^ and 1385–1383 cm⁻^1^ (asymmetric and symmetric C–H stretching in methyl and methylene groups) as well as 1632–1630 cm⁻^1^ (C=O stretching in carboxylate groups) were attributed to the conjugated BA molecules in all BA-loaded MWNT samples [33].

Based on Figure 1a, the classic bands of CS assigned to polysaccharide structures were seen at 3438 cm^−1^ due to the superimposed axial stretching of O–H and N–H groups, and a sharp band observed at 1631 cm^−1^ corresponded to the overlapped axial stretching of C=O in the amide I and N–H deformation of amide II. In addition, the band which appeared at 1384 cm^−1^ can be attributed to the CH_3_ symmetrical angular deformation mode [34]. Additionally, the fingerprint region of glycosidic bonds (C–O and C–O–C stretching) was located over the range of 1050–1030 cm^−1^, and this specific band is extremely advantageous for evidencing the presence of CS even in low amounts [35]. All these bands shifted to a higher wavenumber upon CS coating onto MWBA, indicating the successful conjugation of CS molecules with the MWBA sample. The FTIR spectrum of PG demonstrated broad characteristic bands around 3446, 1104 and 961 cm⁻^1^ due to the O–H stretching, C–O stretching and C–C stretching vibrations, respectively. The bands centered around 2889, 1469, 1344 and 1241 cm⁻^1^ were due to the methylene groups of C-H stretching, bending and twisting vibrational modes. Lastly, the bands that occurred at 961, 842 and 529 cm^−1^ were corroborated to the skeletal vibrations. Comparing the FTIR spectra of pure PG and MWBA-PG, the features related to the vibrational modes of PG were mostly found in the PG-coated nanotubes, with some minor shifting in wavenumbers. These findings confirm that the PG molecules were non-covalently attached to the MWBA formulation.

In the spectroscopic data of pure T2 and T8 (Figure 1b), the absorption bands of O-H stretching detected at 3487 and 3492 cm^−1^ had both shifted to 3435 cm^−1^ in the biopolymeric MWBA nanocomposites. The spectrum exhibited that an intense O-C=O band at 1734 cm^−1^ had shifted to 1723 cm^−1^, and the bands centered around 1092–1094 cm^−1^ corresponding to the stretching vibration of C-O-C had shifted to 1091 cm^−1^. Furthermore, it is interesting to note that the characteristic bands observed in the range of 2921–2856 cm^−1^ which belong to the asymmetric and symmetric methylene stretching vibrations were also seen in the biopolymeric MWBA samples [36]. This observation suggests that the bands could have been overlapped with the C-H stretching of BA molecules, leading to an increase in intensity in the FTIR absorption spectra of MWBA-T2 and MWBA-T8. Overall, the spectra of MWBA-T2 and MWBA-T8 samples exactly resembled pure T2 and T8, as expected since the Tween molecules are derived from the same polysorbate family.

### 3.2. Thermogravimetric Analysis 

**Figure 2:**
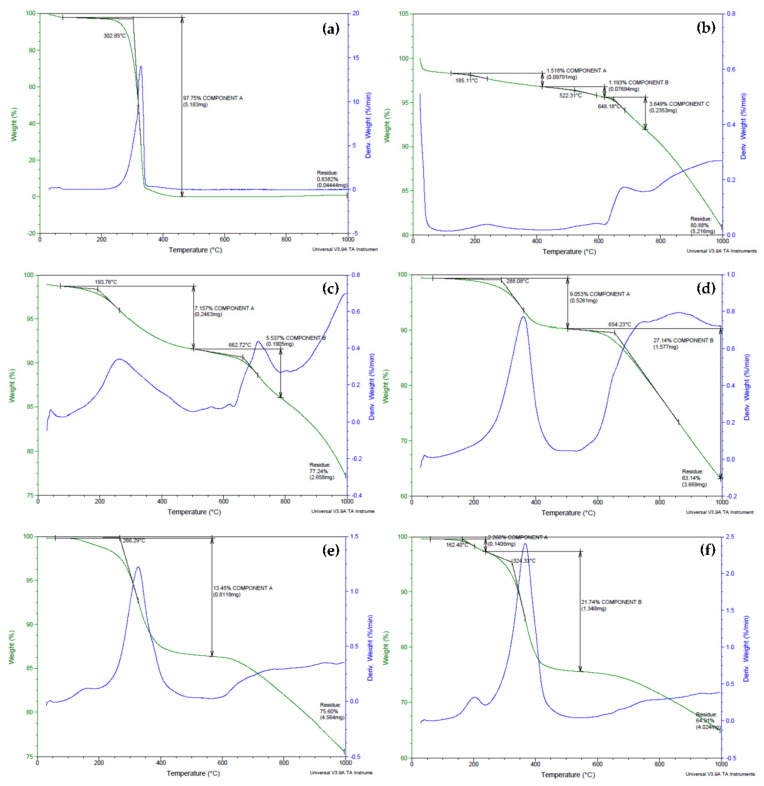
Thermogravimetric analysis (TGA)-DTG thermograms obtained for (**a**) pure betulinic acid (BA), (**b**) MWNT-COOH, (**c**) MWBA-CS, (**d**) MWBA-PG, (**e**) MWBA-T2 and (**f**) MWBA-T8.

Thermal studies using the thermogravimetric analysis (TGA) technique were carried out to investigate the degradation of biopolymeric MWBA nanocomposites in comparison to pure BA and blank MWNT. Based on Figure 2a, pure BA exhibited an intense, single-step thermal decomposition curve at 303 °C that was ascribed to the removal of hydroxyl groups through sublimation, generating a total weight loss of 98%. This strong TGA curve of BA was not detected in the thermograms of biopolymer-coated MWBA nanocomposites, suggesting that the BA no longer existed as a crystalline structure in all four nanocomposites. The obtained result is in line with a TGA study conducted by a team of scientists on the isolation of betulin from birch bark using a vacuum and atmospheric sublimation approach [37]. They reported that the thermal degradation of betulin occurring in the temperature range of 250–370 °C was due to the thermal sublimation of volatile substances. 

As shown in Figure 2b, the thermal property of MWNT-COOH was observed to barely lose any weight from ambient temperature up to 500 °C. This indicates that the oxidized nanotubes were thermally stable up to 500 °C, which is consistent with previous reports published in the literature [19,38]. The TGA thermogram for the degradation of MWNT-COOH illustrates the occurrence of three weight loss steps. The first step, which was related to the evaporation of moisture and water crystallization, occurred at 185 °C (weight loss about 1.5%), and this was followed by subsequent decomposition steps that took place in the temperature range of 522–648 °C (weight loss around 1.2–3.6%). These weight loss steps were due to the degradation of the carboxylic acid functional groups grafted on the nanotubes’ surfaces during the oxidation process. The carboxyl groups reacted to form hydrogen bonds between the positively charged H atom of one carboxyl group and another electronegative O atom from a neighboring carboxyl group, which then developed into a hexagonal hydrogen bond net [39]. This phenomenon leads to an increase in the activation energy and, as a result, the overall thermal stability of the oxidized nanotubes was greatly enhanced. 

The thermograms of the samples presented in Figure 2c–f show that the MWBA-CS, MWBA-PG, MWBA-T2 and MWBA-T8 have similar degradation profiles where two main decomposition stages were observed after the coating treatment was applied. For example, in the temperature ranges between 194–288 and 654–663 °C, approximately 7–9% and 6–27% weight losses were detected for MWBA-CS and MWBA-PG, respectively. On the other hand, MWBA-T2 and MWBA-T8 seemed to demonstrate lower thermal stability at a temperature below 500 °C. For example, about 2% weight loss was recorded at 118–162 °C and about 21% weight loss was seen at 283–324 °C. The first thermal decomposition stage is commonly related to the evaporation of physisorbed water, which was loosely bound to the surface of the sample, while the second stage would be associated with the pyrolytic decomposition of the polymer. The four polymeric drug-loaded nanotubes began to degrade and caused a modest weight loss in the range of 6–27% at a temperature above 260 °C. The weight loss at this temperature was due to the decomposition of the surface functional groups such as O-H, C=O, N-H and C-H. On top of that, all four biopolymer-coated nanocomposites displayed different thermal profiles than the oxidized nanotubes and pure BA, indicating the successful interaction between the MWBA and biopolymer, which subsequently altered the TGA curve of the material. Overall, it was noted that the findings obtained from the TGA study showed a good agreement with the FTIR spectroscopy result, as discussed earlier.

The thermogravimetric property of the samples and their subsequent weight losses in the decomposition stages are summarized in Table 1. Based on the samples’ thermogravimetric characteristics, it was noted that the biopolymeric MWBA nanocomposites demonstrated synergistically improved thermal stability, where the degradation mainly occurred in two steps when compared to pure BA alone. This is because the biopolymer-coated MWBA nanocomposites exhibited a decrease in the particle size due to the addition of nanoscale material acting as a drug delivery agent. Hence, the sample has a higher specific surface area, which eventually led to easier vaporization and earlier decomposition below the temperature of 300 °C. In addition, the interfacial bonds such as hydrophobic and electrostatic forces might have become significantly stronger and more apparent between MWBA and biopolymer after the coating treatment. This can be verified through the increase in the overall effective thermal conductivity of the nanocomposites in the second stage of thermal degradation. This finding is consistent with other similar reports conducted on the thermal properties of nanocomposites derived from polymers and inorganic nanoparticles [40]. 

### 3.3. Raman Spectroscopy

Raman spectroscopy is an essential analytical tool frequently used to characterize carbon-based materials in terms of their chemical structure and physical property. This non-destructive key technique reveals the various characteristic spectra of new nanostructured sp^2^ carbon allotropes, such as carbon fibers, carbon black, pyrolytic graphite, fullerenes, graphenes, CNT as well as their degree of structural disorder [41,42]. 

The Raman spectra of CNT typically consist of a few prominent peaks, namely the G and G’ bands, the D band as well as other relatively weak bands. The G band, or the so-called tangential mode, can be ascribed to the first-order Raman scattering of the degenerate in-plane E_2g_ phonon mode at the Brillouin zone center and normally occurs near 1580 cm^−1^. The disorder-induced D band, which corroborates to the breathing vibrations of the aromatic hexagonal sp^2^ carbon rings, appears around 1350 cm^−1^ and requires a defect for its activation. Hence, the D band intensity relative to the G band intensity (I_D_/I_G_) provides an excellent indicator for showing the structural defects present in carbon nanomaterials [43]. The G’ band, or sometimes also referred to as the harmonic 2D band, is the second-order Raman spectrum due to in-plane transverse optical mode at the K point zone boundary, and normally appears in the range of 2500–2800 cm^−1^. Lastly, the radial breathing mode (RBM), which is related to the atomic vibration of the sp^2^ carbon atoms in the radial direction and is generally observed between 120 and 250 cm^−1^, is the characteristic feature of SWNT.

**Figure 3:**
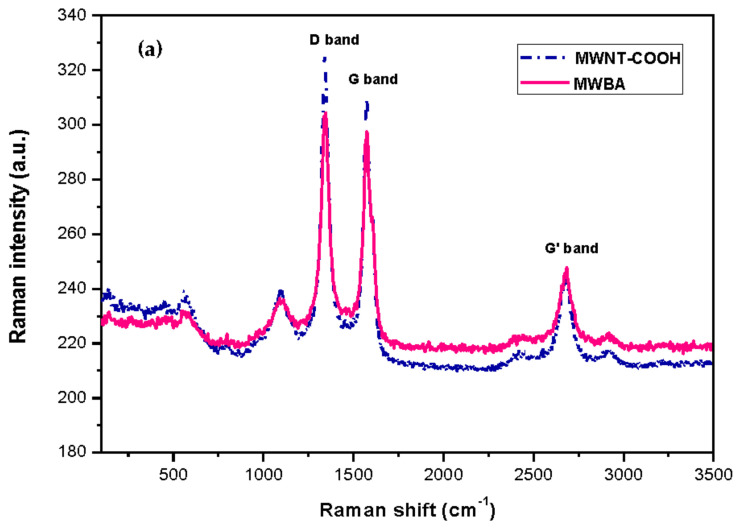

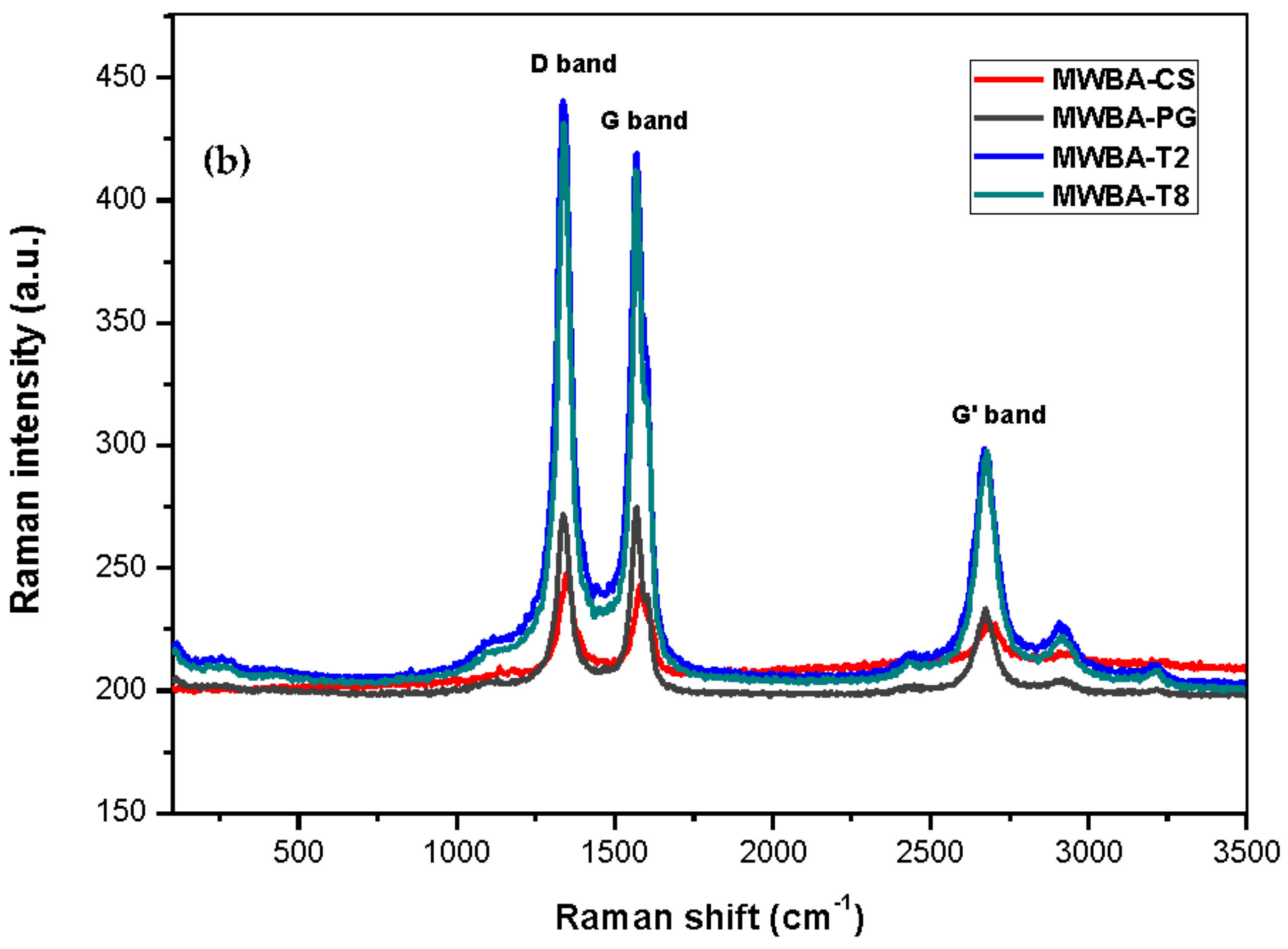
Raman spectra of samples: (**a**) MWNT-COOH, MWBA; (**b**) MWBA-CS, MWBA-PG, MWBA-T2 and MWBA-T8.

Figure 3a illustrates the Raman spectrum of carboxylated MWNT and BA-loaded MWNT for comparison purposes. As can be observed in the Raman spectrum, the main Raman signature bands occurred at 1342 (D band), 1575 (G band) and 2681 (G’ band) cm^−1^. The D band is defect activated Raman mode, suggesting that the samples were not of a complete crystalline graphitic material and that they contained a high amount of defect structures. The presence of the G band (E_2g_ symmetry) confirmed that all samples were composed of sp^2^ carbon networks, which is the essential feature of CNT. The G’ band, which is associated with the stacking order and the number of graphene layers, was clearly seen in the Raman spectrum as a result of interactions between stacked graphene layers. Furthermore, a relatively weak band belonging to the disordered graphitic lattice (A_1g_ symmetry) was seen near 1100 cm⁻^1^. The same band was also observed by other teams, and the authors provisionally assigned it to the stretching vibrations of double and single carbon-carbon bonds in polyene organic compounds or carbon sp^2^-sp^3^ bonds [44,45]. Lastly, the RBM was not detected in the nanotubes as the signals were too weak to be observable. Besides, this characteristic phonon mode is not a common feature for large diameter tubes such as MWNT.

Upon comparison with Figure 3a, it was noted that the Raman bands in the biopolymeric MWBA samples (Figure 3b) became sharper and stronger. In fact, the line width of the bands in all four samples appeared to be much narrower than that of MWBA (uncoated sample). The characteristic bands of MWNT, namely the D, G and G’ bands, were identified at 1338–1346, 1567–1579 and 2671–2674 cm^−1^, respectively, for all four samples as they generally shifted to a lower wavenumber after the coating treatment. When the functional groups belonging to the biopolymer were formed on the surface of nanotubes during the coating process, these signature bands can still be easily identified. This reflects that their structural characteristics were not affected by the application of the biopolymer coating process [46]. In addition, the minor spectral shifts could be due to the successful conjugation of biopolymer molecules with MWBA, where the polymer chains formed a strong interfacial adhesion through σ bond with surface oxygen functional groups [47].

Apart from that, the integrated intensity ratio (I_D_/I_G_) of MWBA with polymer-coated MWBA showed a decreasing fashion from uncoated to coated samples (except for MWBA-T2 and MWBA-T8, where the intensity ratio values were quite similar to the uncoated MWBA). Data obtained from the Raman analysis presented in Table 2 clearly show a reduced defect amount due to the curing effect of the polymer matrix on MWBA when compared to the uncoated MWBA. MWNT-COOH has the highest I_D_/I_G_ ratio among the rest of the samples, indicating that there are more defects as the sp^2^ bonds are broken down and more transition from sp^2^ to sp^3^ carbon. In general, the larger the amount of defects, the higher the D band intensity and, hence, the larger the intensity ratio.

### 3.4. Morphology Study

**Figure 4:**
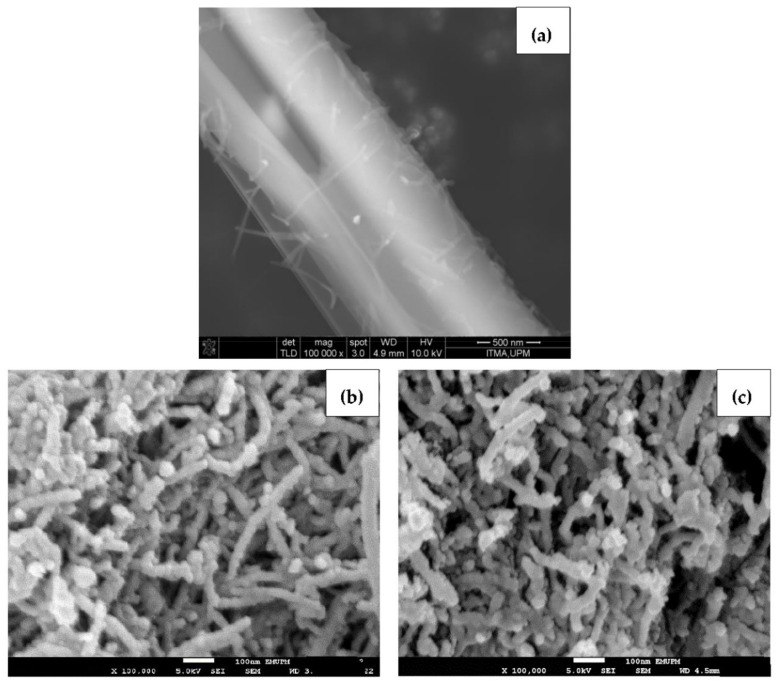
FESEM images of (**a**) pure BA; (**b**) MWNT-COOH and (**c**) MWBA at a magnification of 100,000×.

Figure 4 reveals the surface morphology of pure BA, blank oxidized nanotubes and BA-loaded MWNT. As seen in Figure 4a, the FESEM micrograph shows that a BA crystal appeared as a smooth, elongated microfiber structure with approximately 1 μm in diameter. Figure 4b,c presents the morphology of the MWNT before and after loading with BA. Since the MWBA sample was prepared by the ultra-sonication method, it can be seen that the MWBA nanotubes were in a slightly dispersed physical state while maintaining their tubular nanostructures as compared to the blank nanotubes. Nanotubes are known to easily agglomerate due to strong van der Waals forces and, therefore, they have the tendency to form small bundles. The FESEM observations reflect that the ultra-sonication process enhanced the dispersibility of the nanotubes and, most importantly, it did not damage the tubular shapes of the CNT samples. Although the crystalline structure of BA was no longer visible after it was loaded onto MWNT, the outside diameter of the blank nanotubes was generally seen to increase slightly in thickness from approximately 30 nm to 50 nm after the loading process. These images indicate that the BA molecules were loaded successfully onto the surface of the MWNT, which is in line with the previous analysis results.

**Figure 5:**
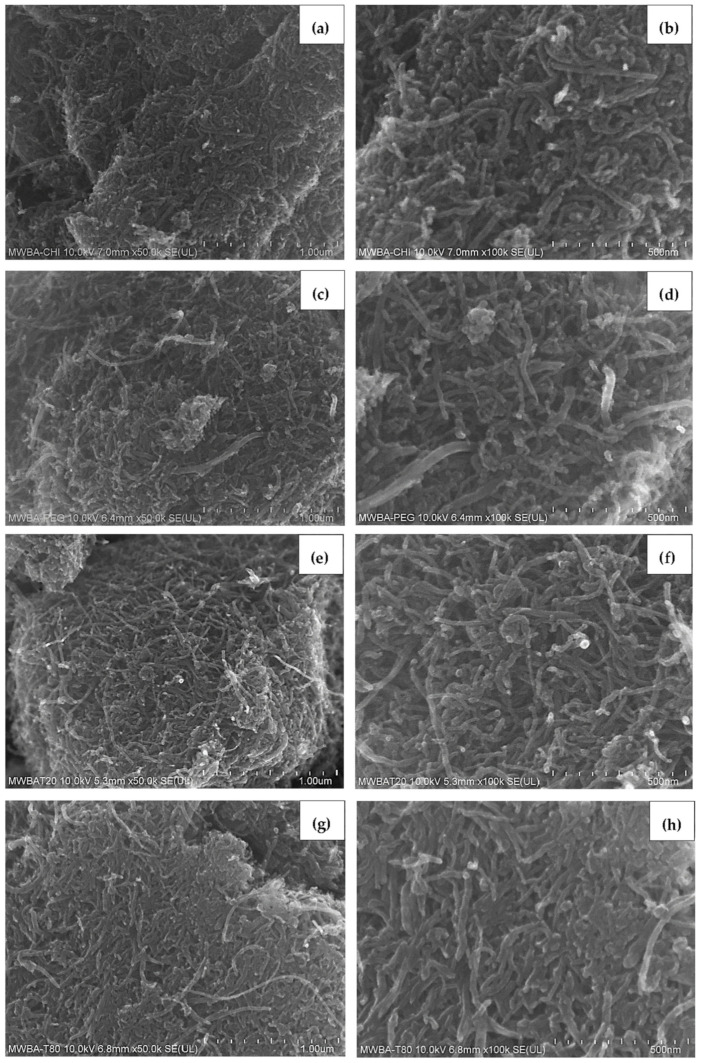
FESEM images of biopolymeric MWBA nanocomposites captured at low (50,000×) and high (100,000×) magnifications: (**a**,**b**) MWBA-CS; (**c**,**d**) MWBA-PG; (**e**,**f**) MWBA-T2; (**g**,**h**) MWBA-T8.

The FESEM characterization analysis of MWBA nanocomposites coated with different types of biopolymers is presented in Figure 5. Based on these images, it was found that the surface morphology of the four samples was flat and more compact due to the formation of the hydrophilic polymer matrix during the coating process. This is more evident especially in Figure 5a,c, where the introduction of the CS and PG molecules through the cross-linking of polymer chains may have improved the interfacial adhesion between the nanotubes and the coating agent. On the other hand, Figure 5e,f demonstrates that the samples were less condensed and more porous, possibly due to the formation of micelles beyond a certain concentration which is known as critical micelle concentration of the surfactants. Overall, it was also revealed that the addition of these biopolymers further decreased the coarse and uneven surface feature of uncoated MWBA, as shown in Figure 4c, through the wrapping technique. This can be verified by the Raman integrated intensity ratio of I_D_/I_G_, where the degree of porosity decreased with the incorporation of the hydrophilic polymer. Furthermore, as seen from the close-up FESEM images, the tubular shapes of the nanotubes were easily identified, suggesting that the surface modification by the layer of biopolymer did not significantly change the nanostructure of the MWNT. 

### 3.5. In Vitro Cytotoxicity Study

Cell culture assay is a very useful technique to assess the biocompatibility of various materials due to its relative simplicity, high sensitivity and good reproducibility for studying the potential toxicity or irritancy of materials and chemicals. To determine the in vitro cytotoxicity effect of the incorporated biopolymer matrix with MWBA, MTT assay was performed according to the literature using a normal and healthy mouse embryonic fibroblast NIH/3T3 cell line [48]. This important fibroblast culture, which was originally established from Swiss mouse embryos, is frequently used in biomedical research for the preliminary screening of materials prior to in vivo assessment [49,50,51]. This cell line provides an ideal way to quantify the cyto-compatibility action of living cells to the nanomaterial owing to its ability to divide indefinitely at a constant growth rate in culture medium, and, most importantly, it is easy to be maintained [52]. 

**Figure 6:**
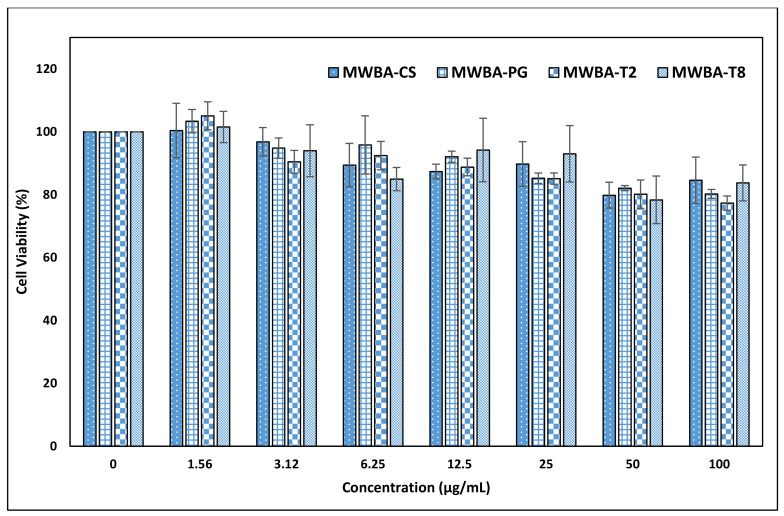
The biological effects of biopolymeric MWBA nanocomposites on NIH/3T3 non-tumor cell line viability after 72 h incubation as assessed by MTT assay. Untreated normal cell lines were used as a control sample. Statistical analysis was performed using one-way ANOVA followed by Tukey HSD post-hoc test (*p* < 0.05).

The observation from our previous study indicated that the percentage of viable cells was found to be reduced substantially to less than 40% when incubated with 50 μg/mL of uncoated MWBA for 72 h [30]. To circumvent this issue, we have decided to coat the MWBA nanocomposites with a layer of hydrophilic polymer to further improve the biocompatibility of the drug formulation on NIH/3T3 cells. The cells acquired from ATCC (Virginia, US) were exposed to varying concentrations (1.56–100 μg/mL) of biopolymeric MWBA nanocomposites, and the results of the MTT assay are presented in Figure 6. The coating agents utilized in this study are natural, highly water-soluble polymer compounds frequently used in food science and biomedical research due to their excellent biocompatibility, low toxicity, good mucoadhesion and stabilizing properties. Subsequently, we have observed that all biopolymer-coated samples did not induce cell mortality when all viable cells were maintained at 80% and above, even at the highest concentration tested (100 μg/mL). This is compelling evidence in which the encapsulated MWBA in the water-soluble polymer matrix exhibited a much higher biocompatibility to the normal cells. The observed data suggest that the coating agent, when incorporated into the formulation, would render the nanomaterial to be a potential drug delivery system in the physiological environment. Nevertheless, further exploration of their cellular uptake and binding mode is still necessary and could prove to be very useful to assess whether this improved formulation could be verified under the in vivo condition.

## 4. Conclusions

In this work, the MWBA nanocomposites were modified by a simple, non-covalent functionalization method with the incorporation of a water-soluble polymer through hydrophobic and electrostatic interfacial bonding. From the results obtained, we found out that the presence of functional groups, which were correlated to the respective polymer molecules, was in good agreement with the thermal decomposition of biopolymeric MWBA nanocomposites and comparable to the decrease in the Raman intensity ratio of those coated samples. The surface morphological study confirmed that the ultra-sonication method did not destroy the tubular structures of the samples, but further improved the dispersion state of the bundled nanotubes. Furthermore, the MTT biological test performed on normal fibroblast NIH/3T3 cells showed no cytotoxic effects at the tested dosages up to 100 μg/mL for all four types of nanocomposites, suggesting their potential use in the application of drug delivery. Even though MTT assay is far superior to other dye exclusion approaches, supplementary control experiments such as WST, lactate dehydrogenase or fluorometric assay should be conducted on par to detect possible chemistry interference when accessing cell viability. Nonetheless, this still warrants a further investigation on the formulation being developed, which requires a higher concentration to be tested on other types of normal cells. 

## Figures and Tables

**Table 1:** The TGA-DTG characteristics of pure BA, pure biopolymers, blank MWNT, MWBA and biopolymeric MWBA nanocomposites.

Samples	Atmosphere	Decomposition Stage		
		First	Second	Third
Pure BA	Weight loss (%) Temperature (°C)	97.8% 302.9 °C	- -	- -
MWNT-COOH	Weight loss (%) Temperature (°C)	1.5% 185.1 °C	1.2% 522.3 °C	3.7% 648.2 °C
MWBA	Weight loss (%) Temperature (°C)	3.2% 153.0 °C	1.1% 537.5 °C	8.3% 636.4 °C
CS	Weight loss (%) Temperature (°C)	48.8% 261.4 °C	- -	- -
MWBA-CS	Weight loss (%) Temperature (°C)	7.2% 193.8 °C	5.5% 662.7 °C	- -
PG	Weight loss (%) Temperature (°C)	42.4% 201.9 °C	57.2% 339.1 °C	- -
MWBA-PG	Weight loss (%) Temperature (°C)	9.1% 288.1 °C	27.1% 654.2 °C	- -
T2	Weight loss (%) Temperature (°C)	95.3% 373.4 °C	- -	- -
MWBA-T2	Weight loss (%) Temperature (°C)	1.5% 117.5 °C	12.0% 266.3 °C	- -
T8	Weight loss (%) Temperature (°C)	96.3% 387.2 °C	- -	- -
MWBA-T8	Weight loss (%) Temperature (°C)	2.3% 162.4 °C	21.7% 324.3 °C	- -

**Table 2:** The relative Raman intensity signature bands for all samples.

**Samples**	**D Band Position** **(cm^−1^)**	**G Band Position** **(cm^−1^)**	**G’ Band Position** **(cm^−1^)**	Intensity Ratio (I_D_/I_G_)
**MWNT-COOH**	1342	1575	2681	1.052
**MWBA**	1346	1575	2685	1.020
**MWBA-CS**	1346	1579	2674	1.008
**MWBA-PG**	1338	1571	2674	0.989
**MWBA-T2**	1338	1571	2671	1.053
**MWBA-T8**	1338	1567	2678	1.046

## Data Availability

The data presented in this study are available on request from the corresponding author.
